# Risk assessment of airborne COVID-19 exposure in social settings

**DOI:** 10.1063/5.0055547

**Published:** 2021-08-20

**Authors:** Chin Chun Ooi, Ady Suwardi, Zhong Liang Ou Yang, George Xu, Chee Kiang Ivan Tan, Dan Daniel, Hongying Li, Zhengwei Ge, Fong Yew Leong, Kalisvar Marimuthu, Oon Tek Ng, Shin Bin Lim, Peter Lim, Wai Siong Mak, Wun Chet Davy Cheong, Xian Jun Loh, Chang Wei Kang, Keng Hui Lim

**Affiliations:** 1 Institute of High Performance Computing, Agency for Science, Technology and Research, 1 Fusionopolis Way, Singapore 138632; 2 Institute of Materials Research and Engineering, Agency for Science, Technology and Research, 2 Fusionopolis Way, Singapore 138634; 3 National Centre for Infectious Diseases, Tan Tock Seng Hospital, 16 Jalan Tan Tock Seng, Singapore 308443; 4 Ministry of Health Singapore, College of Medicine Building, 16 College Road, Singapore 169854; 5 Land Transport Authority, 1 Hampshire Road, Singapore 219428

## Abstract

The COVID-19 pandemic has led to many countries oscillating between various states of lock-down as they seek to balance keeping the economy and essential services running and minimizing the risk of further transmission. Decisions are made about which activities to keep open across a range of social settings and venues guided only by *ad hoc* heuristics regarding social distancing and personal hygiene. Hence, we propose the dual use of computational fluid dynamic simulations and surrogate aerosol measurements for location-specific assessment of risk of infection across different real-world settings. We propose a 3-tiered risk assessment scheme to facilitate classification of scenarios into risk levels based on simulations and experiments. Threshold values of <54 and >840 viral copies and <5% and >40% of original aerosol concentration are chosen to stratify low, medium, and high risk. This can help prioritize allowable activities and guide implementation of phased lockdowns or re-opening. Using a public bus in Singapore as a case study, we evaluate the relative risk of infection across scenarios such as different activities and passenger positions and demonstrate the effectiveness of our risk assessment methodology as a simple and easily interpretable framework. For example, this study revealed that the bus's air-conditioning greatly influences dispersion and increases the risk of certain seats and that talking can result in similar relative risk to coughing for passengers around an infected person. Both numerical and experimental approaches show similar relative risk levels with a Spearman's correlation coefficient of 0.74 despite differing observables, demonstrating applicability of this risk assessment methodology to other scenarios.

## I. INTRODUCTION

Over the past year, COVID-19 has spread worldwide with great speed and intensity causing much loss of life and economic damage. To mitigate the spread of COVID-19, experts have advocated three main interventions. This includes the use of personal protective equipment (PPE), such as masks, face coverings, or face shields, active disinfection of shared places, and minimization of physical interaction via physical distancing guidelines and reduction of large-scale events, such as concerts, conferences, and sporting events.^1–4^ Along those lines, there has been much work on developing more sophisticated PPE and refining the guidance on PPE efficacy.^5–7^

These interventions have generally been effective, with numerous cities and countries observing a substantial reduction in infections upon implementation. Nonetheless, the detrimental impact of lockdown measures on the economy and psychological health of citizens is substantial, including the suspension of activities such as dining at restaurants and participation in large-scale social, religious, or sporting events. Hence, there is a need to re-allow some activities, even if many may end up in close physical proximity.^8,9^ While some general guidelines for risk mitigation exist, including from the World Health Organization, additional science-based analysis of specific scenarios can be helpful for both risk assessment and subsequent customization of mitigating measures.^10^ This is especially important as the specter of a second wave of infections is a persistent worry, and outbreaks upon loosening of restrictions have indeed occurred in several countries spanning Asia and Europe.^11–13^ There has thus been significant modeling of various strategies to better understand and mitigate the risks from re-opening.^14–16^

As more evidence surfaces for the airborne transmission of COVID-19, especially in indoor environments such as restaurants and buses, there has been increasing recognition of the need for better estimates of the risk of infection across different settings.^17,18^ Many have proposed the use of a well-mixed assumption as per the Wells–Riley model, and these can yield insights into the average risk associated with different scenarios.^19–23^ However, a well-mixed model inherently neglects location-specific spatial inhomogeneities, and a more fine-grained analysis is helpful for identifying specific spatial arrangements within the same location that might be of higher risk.

Computational fluid dynamics (CFD) has been widely used to better understand the fundamental physics driving airborne viral transmission.^24–27^ Many have also utilized techniques such as CFD for the analysis of a variety of locations such as clinics, supermarkets, cars, buses, and restaurants.^17,28–34^ In contrast to general guidelines and recommendations, such methods can account for local effects and are useful for identifying higher-risk scenarios and assessing potential mitigation measures to guide re-opening and decision-making. This has provided deep insights into the risk of transmission and evaluation of mitigation measures in diverse scenarios ranging from indoor conference rooms and classrooms to airplane cabins.^35–38^ In this work, we propose a dual use of numerical simulations and surrogate aerosol experiments to study specific social scenarios or mass gatherings in the urban environment. Explicit representation of potential receivers and the source (infectious individual) is used for the analysis of local dispersal patterns and assessment of relative risk of infection. This is achieved by explicit modeling of multiple humans in CFD and placement of a smoke generating machine and multiple aerosol sensors across the domain of interest in the surrogate aerosol experiments.

Importantly, many authors have noted the need for a more intuitive way of comparing transmission risk and communicating results to stakeholders, including policy-makers, in a more interpretable and actionable form.^39,40^ Hence, we further propose a 3-tiered risk assessment scheme based on results from CFD and surrogate aerosol experiments. This also builds on prior proposals for a more nuanced implementation of risk mitigation measures such as physical distances.^41^ This 3-tiered risk scheme can more finely distinguish between higher- and lower-risk scenarios and quantify the effectiveness of various mitigation measures under scenario-specific conditions. Such a risk assessment scheme can also facilitate a more systematic and integrated approach to COVID-19 risk management via a systematic quantitative risk assessment (QRA) style framework, as per the emergency response planning guidelines (ERPG) 1/2/3 bands in the occupational health and safety domain and quantitative microbial risk assessment (QMRA) in the food industry.^42–46^ The proposed stratification into tiers is based on currently available literature on COVID-19 infectivity and is further described in Sec. II.

Using a typical public bus in Singapore as a case study, we demonstrate the use of this risk assessment methodology as a simple and easily interpretable framework for the evaluation of relative risk of infection across different scenarios. The relative risk of infection resulting from different emission activities such as a 1-time cough and talking for 5 min and different positions of the infected passenger are compared under this methodology. The latter comparison, in particular, is not obtainable under the well-mixed assumption. The relative risk levels obtained from both numerical and experimental approaches are further compared and show good consistency.

## II. METHODS

### A. Computational fluid dynamics simulation

#### 1. Theoretical model for airflow and droplet transport

Real-world scenarios are usually very complex and comprise multiple physics such as mass and heat transfer for the entire computational fluid domain, droplet evaporation, and motion of the individual simulated cough droplets. A brief description of the theoretical model used is included here.

The governing equations for fluid mass and momentum are the Reynolds-averaged Navier–Stokes equations,

$$\frac{\partial \rho }{\partial t}+\nabla \cdot \left(\rho \vec{u}\right)=\dot{m,}$$ (1)

$$\frac{\partial \left(\rho \vec{u}\right)}{\partial t}+\nabla \cdot \left(\rho \vec{u}\vec{u}\right)=-\nabla P+\nabla \cdot \left[\mu_{\mathrm{eff}}\left(\nabla \vec{u}+\nabla {\vec{u}}^{T}-\frac{2}{3}\nabla \cdot \vec{u}I\right)\right]+F_{m}+\rho \vec{g}\beta \left(T-T_{0}\right).$$ (2)

The source terms in Eqs. (1) and (2) account for mass transfer between the air and droplet due to evaporation. *μ*_eff_ is the effective viscosity considering both dynamic and turbulent viscosities. In addition, the realizable κ-ε turbulence model is used for the modeling of *κ,* the turbulent kinetic energy, and *ε,* the dissipation rate of turbulent energy, as described in Eqs. (3) and (4),

$$\frac{\partial \left(\rho \kappa \right)}{\partial t}+\nabla \cdot \left(\rho \vec{u}\kappa \right)=\nabla \cdot \left(\left(\mu +\frac{\mu_{t}}{\sigma_{k}}\right)\nabla \kappa \right)+G_{k}+G_{b}-\rho \varepsilon ,$$ (3)

$$\frac{\partial \left(\rho \varepsilon \right)}{\partial t}+\nabla \cdot \left(\rho \vec{u}\varepsilon \right)=\nabla \cdot \left(\left(\mu +\frac{\mu_{t}}{\sigma_{k}}\right)\nabla \varepsilon \right)+\rho C_{1}S\varepsilon -\rho C_{2}\frac{\varepsilon^{2}}{\kappa +\sqrt{\nu \varepsilon }}+\frac{\varepsilon }{\kappa }C_{1\varepsilon }C_{3\varepsilon }G_{b}.$$ (4)

The standard model constants, *C_1ε_* and *C_2_*, are 1.44 and 1.9, respectively, while *σ_κ_* and *σ_ε_* are 1.0 and 1.2, respectively. *G_k_* and *G_b_* are the production of turbulent kinetic energy due to mean velocity gradients and buoyancy, respectively. The eddy viscosity *μ_τ_* is given by the following expression:

$$\mu_{t}=\rho C_{\mu }\frac{\kappa^{2}}{\varepsilon }.$$ (5)

In addition, to account for droplet evaporation and consequent changes in the droplet size, the following species transport equations are solved for the different constituent species in air, modeled in this work as nitrogen, oxygen, and water vapor:

$$\frac{\partial \left(\rho x_{i}\right)}{\partial t}+\nabla \cdot \left(\rho \vec{u}x_{i}\right)=-\nabla \cdot {\vec{J}}_{i}+S_{i},$$ (6)

where ${\vec{J}}_{i}$ is the diffusive flux of species *i* as given by

$${\vec{J}}_{i}=-\left(\rho D_{i,m}+\frac{\mu_{t}}{Sc_{t}}\right)\nabla x_{i}-D_{t}\frac{\nabla T}{T},$$ (7)

where $Sc_{t}$ is the turbulent Schmidt number (specified as 0.7) and $D_{t}$ is the thermal diffusivity. The source term for each species, $S_{i}$, is zero for nitrogen and oxygen and non-zero for water vapor when droplet evaporation is occurring.

Heat transfer within the computational domain is accounted for by the following energy conservation equation:

$$\frac{\partial \left(\rho E\right)}{\partial t}+\nabla \cdot \left(\rho \vec{u}E\right)=\nabla \cdot \left(\left(\lambda \nabla T\right)-\sum_{i}h_{i}{\vec{J}}_{i}\right)+S_{h},$$ (8)

where *λ* is the effective thermal conductivity which considers both thermal conductivity of air, *λ_a_,* and turbulent thermal conductivity, *λ_t_*. *λ_t_* and E are given by

$$\lambda_{t}=\frac{c_{p}\mu_{t}}{{Pr}_{t}},$$ (9)

$$E=h-\frac{P}{\rho }+\frac{{\vec{u}}^{2}}{2},$$ (10)

where *μ*_t_ is the eddy viscosity, Pr_t_ is the turbulent Prandtl number which is set to 0.85, and h is the sensible heat as given by

$$h=\sum_{i}x_{i}C_{p,i}T+\frac{P}{\rho },$$ (11)

and $S_{h}$ is the thermal source term due to droplet motion and evaporation.

Droplet evaporation is modeled by diffusive molar flux of water vapor into the air, *N_v_*, via the following expression:

$$N_{v}=k_{c}\left(C_{vd}-C_{va}\right),$$ (12)

where *C_va_* is the vapor concentration in air and *C_vd_* is the vapor concentration at the saturated vapor pressure *P_sat_* on the droplet surface.

*C_vd_* is related to *P_sat_* by the following:

$$C_{vd}=\frac{P_{\mathrm{sat}}}{RT_{d}},$$ (13)

where *T_d_* is droplet surface temperature.

*C_va_* is computed from partial vapor pressure by the following expression:

$$C_{va}=x_{v}\frac{P}{RT_{a}},$$ (14)

where $x_{v}$ is the species molar fraction and *P* and *T_a_* are the local pressure and temperature, respectively.

The mass transfer coefficient *k_c_* is correlated with Reynolds number (*Re*) and Schmidt number (*Sc*) by the following expression:

$$k_{c}=\frac{D_{dv}}{D_{d}}\left(2.0+0.6{Re}^{0.5}{Sc}^{1/3}\right),$$ (15)

where *D_dv_* is the diffusion coefficient of vapor in the air. The mass of the droplet evolves with time as

$$\frac{dm_{d}}{dt}=-N_{v}A_{d}M_{d},$$ (16)

where $m_{d}$ is the droplet mass, *M_d_* is the molecular weight, and *A_d_* is the droplet surface area.

The droplet temperature is governed by

$$m_{d}C_{pd}\frac{dT_{d}}{dt}=hA_{d}\left(T_{a}-T_{d}\right)-\frac{dm_{d}}{dt}h_{fg},$$ (17)

where *h_fg_* is the latent heat of the droplet. The convective heat transfer coefficient *h* is calculated via the use of a modified Nusselt number,

$$h=\frac{\lambda ln\left(1+B_{T}\right)}{D_{d}B_{T}}\left(2+0.6{Re}_{d}^{0.5}{Pr}^{1/3}\right),$$ (18)

where *Pr* is the Prandtl number, *B_T_* is the Spalding heat transfer number, and *D_d_* is the droplet diameter.

The equation of motion of each droplet is given by

$$\frac{d{\vec{u}}_{d}}{dt}=-F_{D}\left({\vec{u}}_{d}-\vec{u}\right)+\frac{\vec{g}\left(\rho_{d}-\rho \right)}{\rho_{d}},$$ (19)

${\vec{u}}_{d}$ and $\vec{u}$ are the droplet and air velocities, respectively, while $F_{D}$ is the inverse of droplet momentum relaxation time as given by

$$F_{D}=\frac{18\mu }{\rho_{d}D_{d}^{2}}\frac{C_{D}Re}{24},$$ (20)

where *C_D_* is the Re-dependent drag coefficient as given by

$$C_{D}=c_{1}+\frac{c_{2}}{Re}+\frac{c_{3}}{{Re}^{2}},$$ (21)

where *c_1_*, *c_2_*, and *c_3_* are Re-dependent constants for spherical droplets.

#### 2. Computational setup

A polyhedral unstructured scheme was used for meshing all the scenarios studied using ANSYS FLUENT 2019. The Euler–Lagrange approach was adopted for all simulations. The fluid phase is treated as a continuum phase while the discrete droplets are individually tracked based on a Lagrangian reference frame. The realizable κ-ε model, species transport model, and discrete phase model in ANSYS FLUENT 2019 were used for simulating the domain's mass and heat transfer, individual species distribution, and particle evaporation and trajectory as per previous work.^47^ In general, steady state simulations were first performed to obtain a converged solution for fluid flow and heat transfer as well as the corresponding species concentration for nitrogen, oxygen, and water vapor. Transient simulations of potential droplet trajectories across emission events in the different scenarios were then conducted with a pre-converged steady state solution as the initial condition. The trajectory of each droplet is predicted by iteratively solving for the force balance on the droplet at each time step via two-way momentum coupling between the droplet and continuum phase. In addition, the impact on particle trajectories due to turbulent fluctuations can be significant and is extremely complex, with many sophisticated approaches developed to handle this in the context of RANS models.^48,49^ For this work, the effect of turbulence on particle dispersion is incorporated via the discrete random walk model (DRW) natively available in ANSYS FLUENT.^50^ In this model, the fluctuating velocity components are modeled as discrete piecewise constant functions of time. By integrating the trajectory equation [Eq. (19)] for each particle using the instantaneous fluid velocity along the particle path, the random effects of turbulence on the particle dispersion can be included. Each human in the simulation is explicitly modeled and specified as a heat source with an average surface temperature of 36 °C, while boundary conditions for inflow and outflow from the computational domain are specified according to real-world operating conditions.

A cough droplet size distribution comprising 4897 droplets based on experimental measurements reported by Duguid is used to specify a typical cough for our simulations and reproduced in Table I.^51,52^ These droplets were injected into the domain with a given location, velocity, and mass. As such, the moving trajectory for every single droplet will be captured. A time-varying cough jet flow velocity is specified based on prior literature and assuming a constant mouth opening area of 4 cm^2^.^53^ For talking scenarios, droplets are assumed to be expelled horizontally at a constant velocity of 0.5 m/s. Each droplet is assumed to be composed of 93.5% water and 6.5% nonvolatile components such as salt and proteins by mass.^54^ No breakup is considered due to the low We number (maximum We number is 1.7) for the droplet size range of interest while droplet–droplet interactions are neglected due to their relatively low number density.^55^ All surfaces in the simulation are also assumed to be perfect traps, implying that each droplet is assumed to stick to any surface once it comes into contact with the surface, with no degradation of any existing viral load in the droplet.

**TABLE I.** Droplet size distribution from the experimental study by Duguid.^42^

Diameter (*μ*m)	2	4	8	16	24	32	40	50
Number	44	284	966	1592	865	415	235	105
Diameter (*μ*m)	75	100	125	150	200	250	500	1000
Number	136	79	43	33	31	27	29	13

In general, a wide range of viral loads in respiratory droplets has been reported over the course of disease progression, with variation across multiple orders of magnitude.^56^ An intermediate range of 10^8^ viral copies per ml is used as a baseline for risk assessment, and the viral concentration is assumed to be constant across all droplet sizes. Also, the independent action hypothesis, which assumes that individual viral copies across all the different droplets are equivalent in infectivity, is presumed to be valid for COVID-19 for this methodology. Details can be found in the work by Li *et al.*^57^

### B. Surrogate aerosol experiment

In order to simulate aerosol emission by humans, a LED-500 fogging machine was used to generate aerosols with a mean particle size of approximately 1 *μ*m in diameter. A mixture of 50% water and 50% glycerol was used as the fogging liquid. Particulate sensors (Sensirion SPS30) were used to detect and quantify the fog concentration. The sensors were calibrated using a TSI DustTrakTM DRX Aerosol Monitor 8533 as a reference. The particle sensors were tested for linearity and calibrated for 1 *μ*m size aerosol to ensure data accuracy. The time-dependent aerosol concentration was continuously measured at a sampling rate of 1 Hz, with real-time data sent wirelessly to a computer for logging. In addition, to account for any variability in emission from the fog generator, a 5-min time-averaged measurement is recorded for each experiment. More details on the experimental setup can be found in our earlier work along with a more comprehensive review by Su *et al.* on various technologies for studying air quality.^58,59^ To quantify the cumulative concentration of aerosol emitted from the fog generator, a reference sensor is always placed right in front of the opening of the fog generator. The remaining sensors are placed at various distances and directions, depending on the test scenario of interest.

### C. Risk-level determination

#### 1. Risk-level determination by CFD simulations

Since the early stages of the COVID-19 pandemic, there have been numerous investigations into the different physiological aspects of the disease, such as variation in viral shedding with disease progression, the extent to which viral shedding (or droplet emission) varies with activity, and infectivity of COVID-19.

Current literature in relation to infectivity is still lacking, especially in view of the ethical issues associated with human experimentation. Nonetheless, Schroder reported that infectious doses (ID_50_) of 280 and 790 viral copies were estimated for SARS-CoV-1 and Influenza A, respectively, with Mittal *et al.* estimating a minimum infectious dose on the order of 1000 virions based on these other related illnesses, while Basu estimated a dose of approximately 330 viral copies based on an analysis of the Washington Chorale event.^40,60,61^ Similarly, Buonanno *et al.* used values of between 10 and 100 viral copies as being sufficient for infection when estimating the risk across different scenarios, based on prior literature on SARS-CoV-1,^22^ while Yang *et al.* proposed values of between 100 and 1000 as the characteristic dose.^62^ Other animal studies regarding the infectivity of COVID-19 on hamsters, macaques, and ferrets are summarized in a review paper by Karimzadeh *et al.*, with the lowest infective dose of 1000 plaque-forming units (PFUs) reported for hamsters.^63^ A similarly large range is reported across different influenza strains in humans, ranging from 0.4 PFU for H2N2 to 7 × 10^6^ PFU for H3N2.

In addition, some papers have emerged recently showing that viral RNA can be sampled in air and can even be cultured.^64–67^ In particular, Chia *et al.* reported measuring 3384 viral copies per m^3^ of air from their samplers placed at 1 m distance from a patient with a polymerase chain reaction (PCR) test cycle threshold (C_t_) value of 18.45, which suggests that the patient is potentially highly infectious. Persons in close contact with an infected person for more than 30 min are generally considered to be at high risk of being infected.^68^ Hence, utilizing typical estimates for a tidal volume of 0.5 l and a breathing rate of 16 breaths/min, a second person at a 1 m distance from the patient would have inhaled approximately 840 viral copies within a 30 min period (using an estimate of 3500 viral copies per m^3^ of air). Hence, we propose using this as a threshold for estimating that an individual is at high risk of being infected.

On the other end of the spectrum, Chia *et al.* were unable to detect any viral RNA in the air when sampling a patient in the second week of the disease (C_t_ = 33.22), while C_t_ values of 34 and above, when sampled directly from patients, have been reported to be indicative of a patient that is no longer infectious.^65,69^ Utilizing work by Yu *et al.*, a C_t_ value of 34 in a swab sample is estimated to correspond to 58 viral copies, which is expected to be insufficient for infection of a new person.^70^

**FIG. 1.**
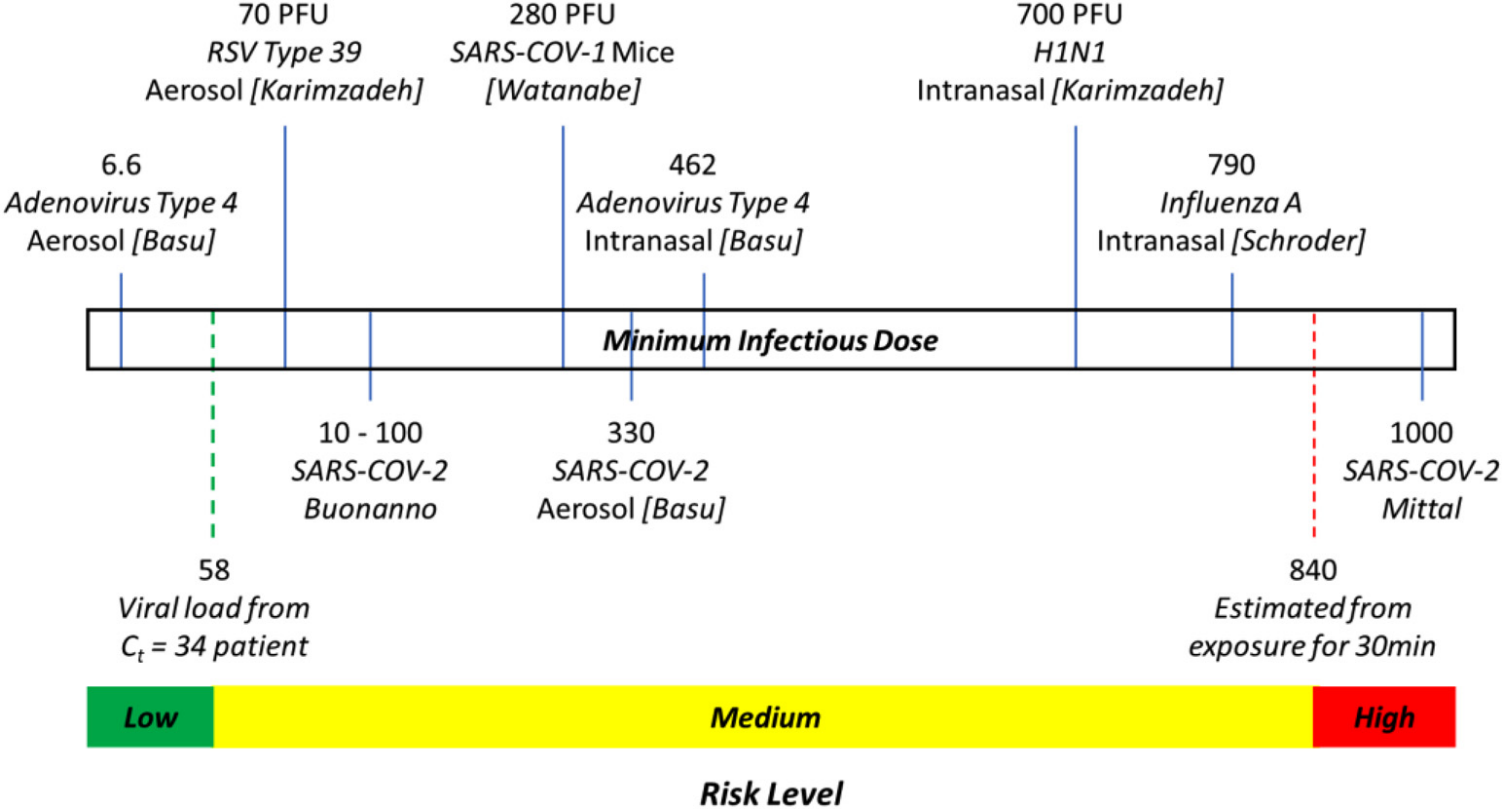
Summary of 3-tier thresholds for risk assessment as used in this work, with selected animal and human studies of minimum infectious dose for related diseases. Widths of the green, yellow, and red ranges are not drawn to scale.

Hence, we propose the use of a 3-tiered risk system, as illustrated in Fig. 1, whereby exposure to less than 58 viral copies in our CFD simulation corresponds to a low risk of infection and exposure to more than 840 viral copies corresponds to a high risk of infection. This range is also consistent with previous estimates for infectivity of approximately 300 as proposed by Basu and other ranges commonly proposed in current literature of between 10 and 1000.^22,40,61,62^ Hence, this risk stratification is proposed for use in the analysis of CFD simulations of specific worst-case scenarios.

In the subsequent analysis, simulated droplet trajectories are traced in the transient CFD simulations, and their final destinations are analyzed. Using the conversion factor of 10^8^ viral particles per ml, the amount of viral load on each surface can be obtained. The relative amount of viral load resulting from a person coughing or talking is then computed and segregated by the risk level for the scenarios studied.

#### 2. Risk-level determination by surrogate aerosol experiments

**FIG. 2.**
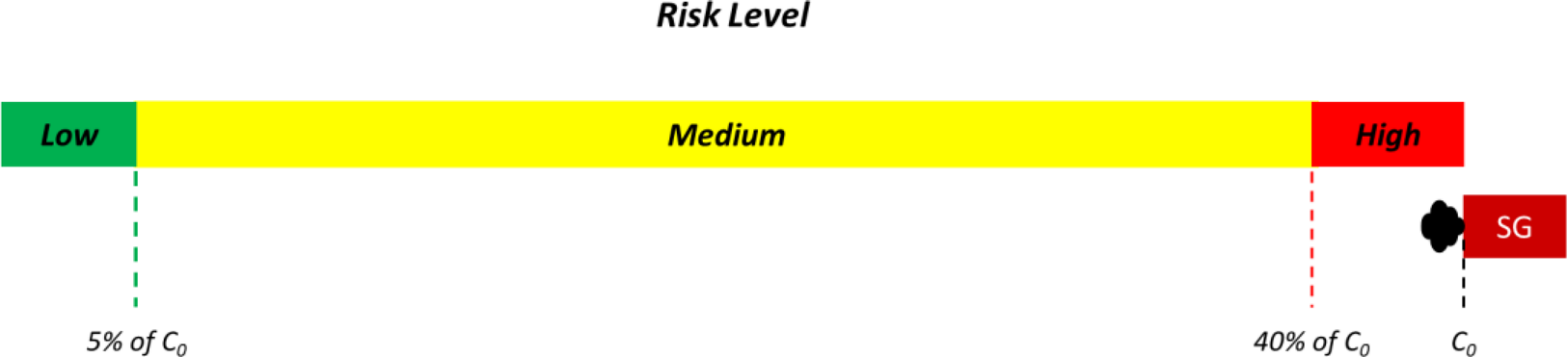
Summary of 3-tier thresholds for risk assessment as used in conjunction with surrogate aerosol experiments. C_0_ is the aerosol concentration as emitted from the smoke generator (SG), which serves in these experiments as a surrogate source. Widths of the green, yellow, and red ranges are not drawn to scale.

Similar to risk level assessment by CFD simulations, a 3-tiered risk assessment scheme is proposed for improving the interpretability of surrogate aerosol experiments. In this instance, low, medium, and high risks are defined using relative aerosol exposure of <5%, 5%–40%, and >40% of the emission source, respectively, as depicted in Fig. 2. As the measured absolute values of aerosol concentration will be dependent on the smoke generator (SG), the relative reduction in aerosol concentration is normalized by the aerosol concentration as emitted by the smoke generator (C_0_). The 3-tiered risk classification is based on estimates for the relative decrease in droplet concentration and consequently, exposure probability, across various distances by Sun and Zhai.^20^ Hence, measured aerosol concentrations of >40% of C_0_ is chosen to represent high risk due to Sun *et al.* showing that this relative reduction corresponds to the exposure level at a distance of 1 m, which is the current minimum distance prescribed for social distancing. Similarly, the measured aerosol concentration of <5% of C_0_ is chosen as it corresponds to aerosol exposure levels at a distance of >8 m. In addition, 5% also corresponds to the N95 mask filtration efficiency, use of which is typically considered to render contact with an infected patient relatively low risk. The risk level across various locations and distances is thus defined based on the reduction in the concentration of surrogate aerosols detected by the particulate sensors at each location relative to the emission concentration.

The use of relative concentration of surrogate aerosols for risk assessment does however rely on the assumption that each aerosol contains the same concentration of viral load and can be treated as being equivalent. Nonetheless, in the absence of further experimental studies to verify biological variations in viral load, relative reductions in surrogate aerosol concentration are proposed as a means of quantifying the relative reduction of risk for various mitigation measures. In order to focus on the risk from relatively smaller, potentially fully evaporated droplet nuclei, surrogate aerosols of 1 *μ*m diameter were used in our study, which is also consistent with prior literature which reported that human coughs typically generate an average droplet nuclei size of between 0.74 and 2.12 *μ*m upon fully evaporating^71^ while talking can generate average droplet nuclei sizes of approximately 4 *μ*m.^72^

### D. Risk assessment of passengers in a public bus

In order to demonstrate the use of this risk assessment methodology, we conducted both CFD and experimental modeling of a simulated infected individual among the passengers seated in a typical double-decker public bus and assessed the relative risk across different scenarios in this work. This scenario was particularly interesting as prior literature had already suggested the potential for transmission of COVID-19 in public transport, including buses, in the absence of any precautions such as face masks or forced ventilation.^18,73^

**FIG. 3.**
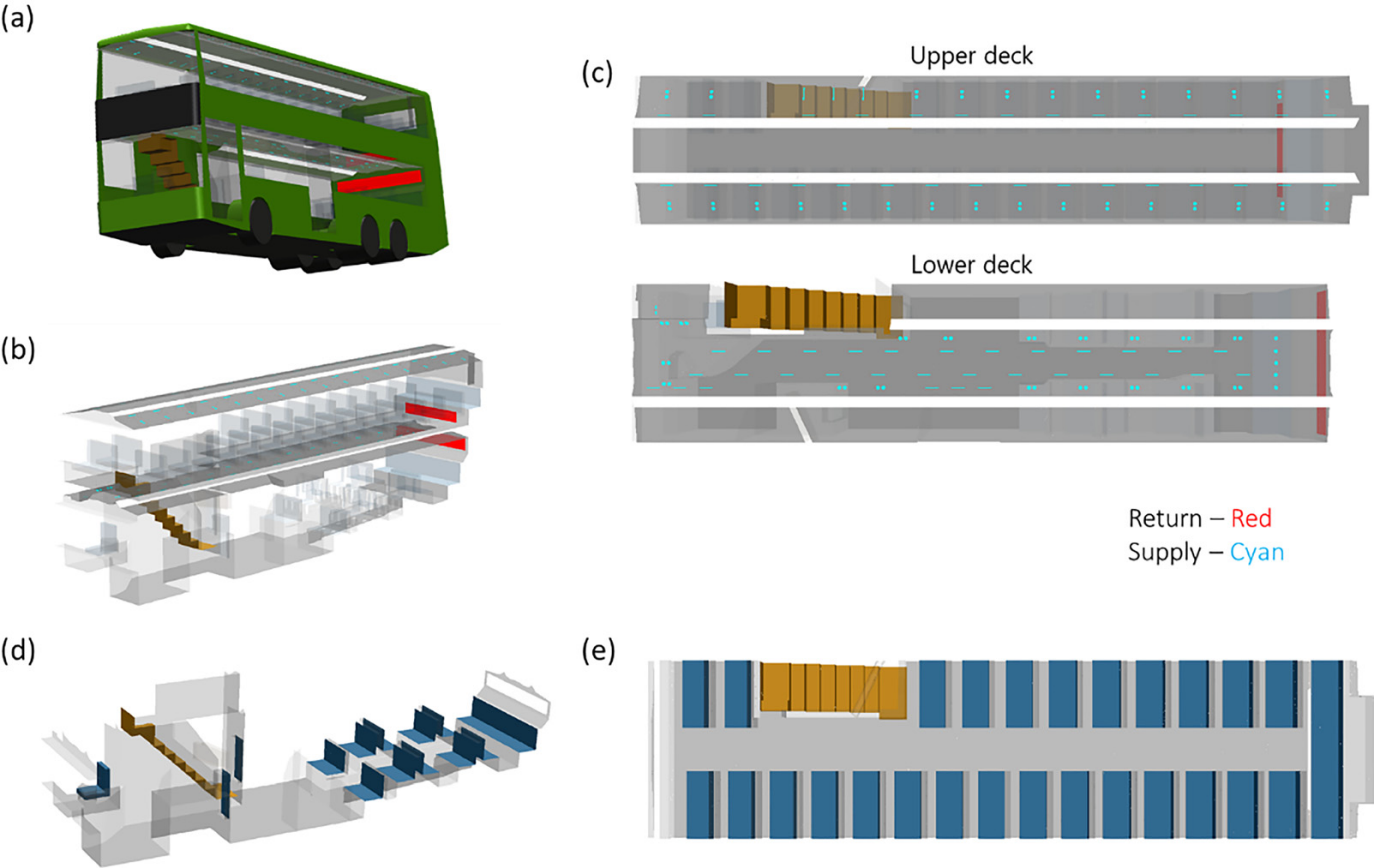
(a) Model re-creation of a typical double-decker bus based on as-built drawings and actual measurements. (b) Isometric view of the air-conditioning supply and return locations in the bus. (c) Top-down view of the air-conditioning supply and return locations in the bus for the upper and lower deck. (d) Isometric view of the seat layout in the lower deck of the bus. (e) Top-down view of the seat layout in the upper deck of the bus.

A simplified geometry of the bus was created as the computational domain for the CFD simulations based on supplier-provided drawings and pictures. The CFD model for the double-decker air-conditioned bus has internal dimensions of 2.5 (width) × 11.9 (length) × 4.1 m (height) as depicted in Fig. 3. Multiple air-conditioning vents (eyeball type) and air-conditioning louvers are distributed across the ceiling, and a large return duct is located near the back of the bus for both the upper and lower decks. The bus is maintained at between 22 and 25 °C and 60% relative humidity during operation and was kept at similar values in both simulation and experiment. The entrance and exit on the lower deck are closed during normal operation and were kept closed for both simulations and experiments.

The double-decker bus is split into two decks, with the bus doors located on the left side of the lower deck, and a set of stairs to the upper deck on the right side of the lower deck. Individual seats are modeled as 0.45 m wide, with a seat depth of 0.45 m. The rear of the lower deck contains five alternating rows of front- and rear-facing seats while the last row is composed of five seats across the entire width of the bus. The seats on the upper deck are identical to the lower deck in dimensions and are composed of 15 rows of seats on the left side and 11 rows of seats on the right side. Schematics of the seat layout are provided in Fig. 3 for clarity.

In general, it is accepted that the use of PPE can greatly reduce the risk of infection, with several studies demonstrating the efficacy of face masks.^74–76^ While PPE use will be mandated in many instances, it is still informative to consider possible different worst-case scenarios. Hence, we choose a first set of simulations and experiments to illustrate how the risk assessment methodology can be applied to different scenarios where PPE is not being properly used. This includes scenarios such as when an infected person coughs or talks when his or her mask is temporarily removed or displaced. A total of three possible emission scenarios are studied and assessed, comprising talking without a mask, coughing without a mask, and coughing with a poorly worn mask. In particular, the infected person is assumed to be talking for 5 min and emitting droplets at a constant rate throughout, whereas the cough scenarios assume a single cough that occurs at the very start of the simulation and experiment.

**FIG. 4.**
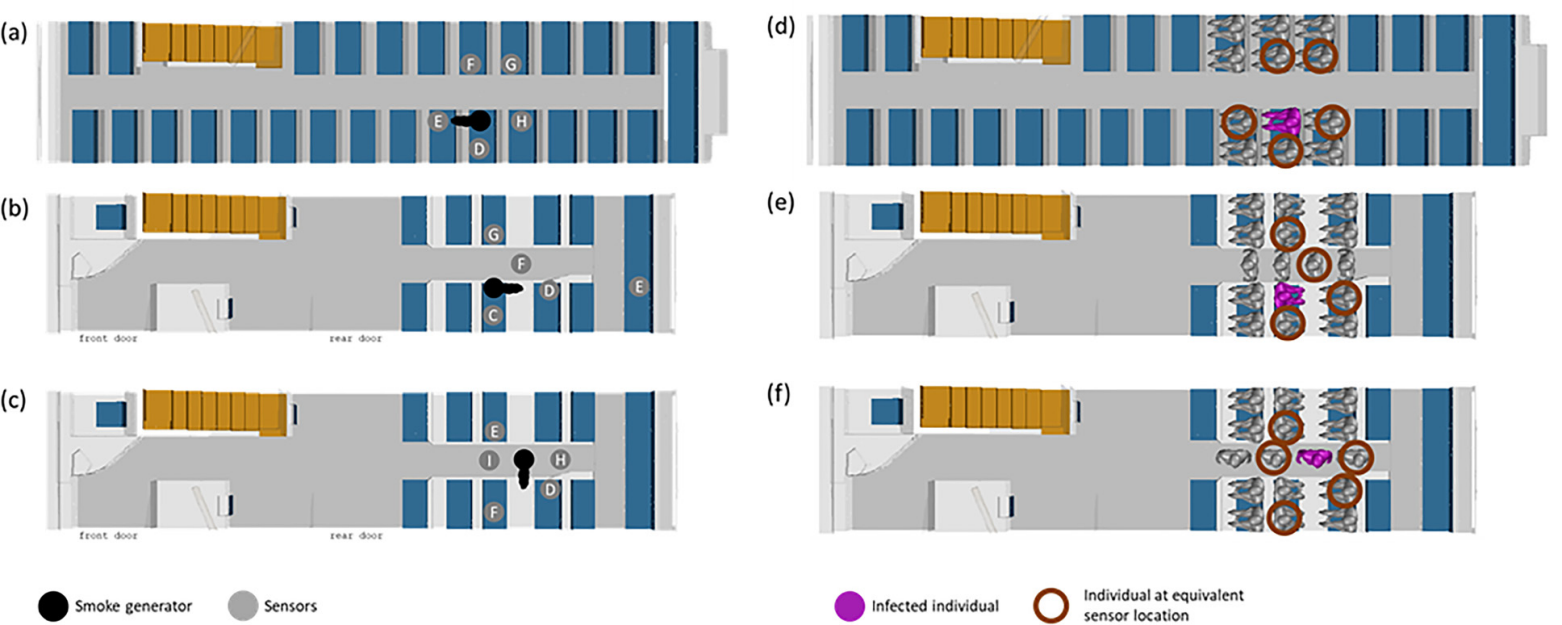
Illustration of the scenarios evaluated via our proposed relative risk assessment framework. Three arrangements (cases A–C) are tested, with the corresponding surrogate aerosol schematic depicted in (a)–(c) and the CFD schematic depicted in (d)–(f). The “infected individual” is depicted in black for (a)–(c) and magenta for (d)*–*(f).

In addition, while the well-mixed assumption is common for general guidelines, our proposed risk assessment methodology can provide more fine-grained, location-specific information. Hence, we further demonstrate the use of this risk assessment methodology by evaluating the relative risk associated with three common positions for a potentially infected and coughing passenger: (A) the passenger is seated and facing forward, (B) the passenger is seated and facing backwards, and (C) the passenger is standing and facing sideways. Schematics of these three scenarios are provided in Fig. 4. The location of the emission source is demarcated in black (smoke generator as used in the surrogate aerosol experiments) or magenta (infected individual as simulated in CFD). A summary of the scenarios is provided in Table II for clarity.

**TABLE II.** Summary of different scenarios on which the proposed risk assessment methodology is demonstrated.

	Case A: Seated passenger facing forward on upper deck	Case B: Seated passenger facing backward on lower deck	Case C: Standing passenger facing sideways on lower deck
Case 1: Coughing without mask	Scenario A1	Scenario B1	Scenario C1
Case 2: Coughing with poorly worn mask	Scenario A2	⋯	⋯
Case 3: Talking without mask for 5 min	Scenario A3	⋯	⋯

**FIG. 5.**
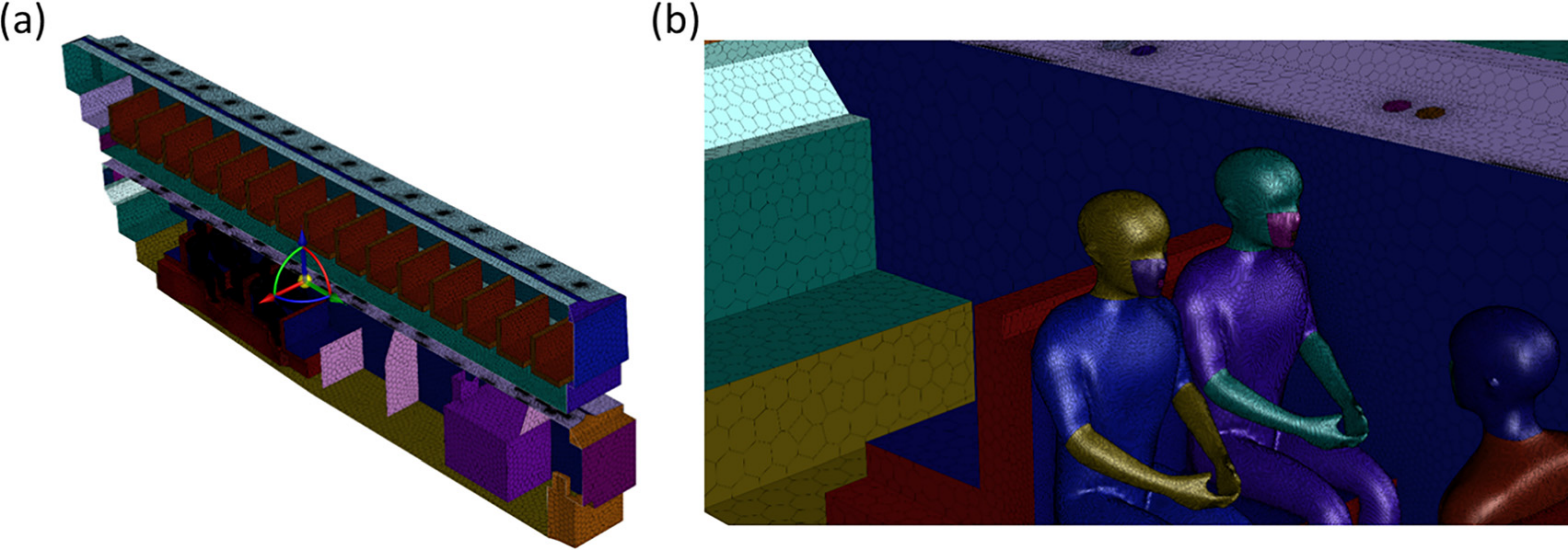
(a) Illustration of the surface mesh resolution across the entire bus. (b) Illustration of the surface mesh resolution used for the seats and people in the region of interest.

The mesh size is approximately 6.1 × 10^6^ cells for case A and 8.6 × 10^6^ cells for cases B and C. Sizing functions (which control the mesh size and curvature) were used to efficiently control the grid density in space and permit fine mesh resolution near the region of interest. For example, our meshing strategy resulted in a maximum grid size of 2 and 5 mm on the mouth and body of individuals in the domain and maximum grid size of 5 cm for the surrounding seats. Similarly, fine mesh settings of between 2 and 5 mm were applied at the air supply and return of the HVAC system. The spatial volumetric mesh sizing was subsequently restricted to gradually increase from the surface mesh at a maximum expansion rate of 1.2× during the volumetric element generation. Inflation layers of volumetric elements were also extruded along the normal direction of bounded walls to better resolve boundary layer effects. Through this treatment, we could preferentially create a significantly denser mesh in the region surrounding the people and seats in order to better resolve the flow features in regions of interest. Illustrations of the surface mesh used for the bus, seats, and people are provided in Fig. 5.

The flow rate of each supply vent or louver was specified based on average values of approximately 3.6 m/s as measured from an actual bus. The lower deck return duct was divided into five uniform flow regions with an area weighted average velocity of 1.9 m/s based on measurements at the corresponding locations from an actual bus and a zero-pressure outlet boundary condition for the center region of the return duct. A uniform flow profile was also applied for the upper deck return. In the CFD simulations, additional passengers are included in the simulation to provide realistic flow obstruction and heat load to reflect typical usage.

**FIG. 6.**
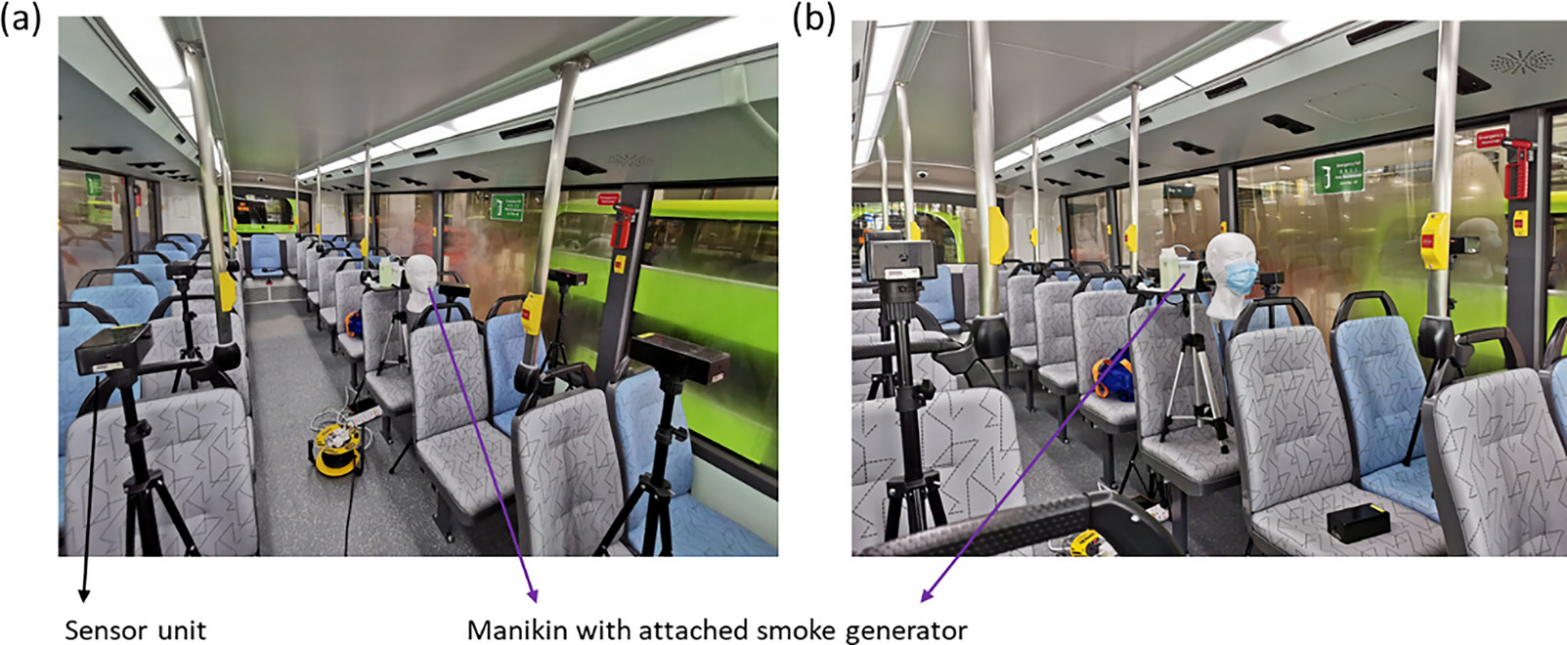
Pictures of a surrogate aerosol experiment showing typical setups with a manikin (a) without and (b) with a face mask, attached to a smoke generator, and accompanying sensor units for assessing relative concentrations at different locations.

In addition, surrogate aerosol experiments were conducted within an actual bus, with the internal air-conditioning system set to typical operating conditions as described above. For each instance, sensors are placed in multiple locations corresponding to different seats in the bus, as depicted in Fig. 4. The smoke generator and sensors are placed at a height of either 1.5 or 1.2 m from the floor to simulate the typical height of a standing or seated passenger. Figure 6 illustrates the placement of smoke generator and sensor units across different experiments.

## III. RESULTS

### A. Assessment of relative risk due to different emitting activities

In such double-decker buses, passengers are likely to choose to sit in one of the two rows of seats facing forwards within the bus. This particular scenario is thus chosen as a case study for assessing the relative risk to others around a potentially infected passenger when the individual is talking for 5 min (case A3) or coughing without a mask (case A1), or with a poorly worn mask (case A2). In this instance, a poorly worn mask is simulated in CFD by a narrow gap between the individual and the mask and in surrogate aerosol experiments by the placement of a mask that does not perfectly fit the human manikin.

**FIG. 7.**
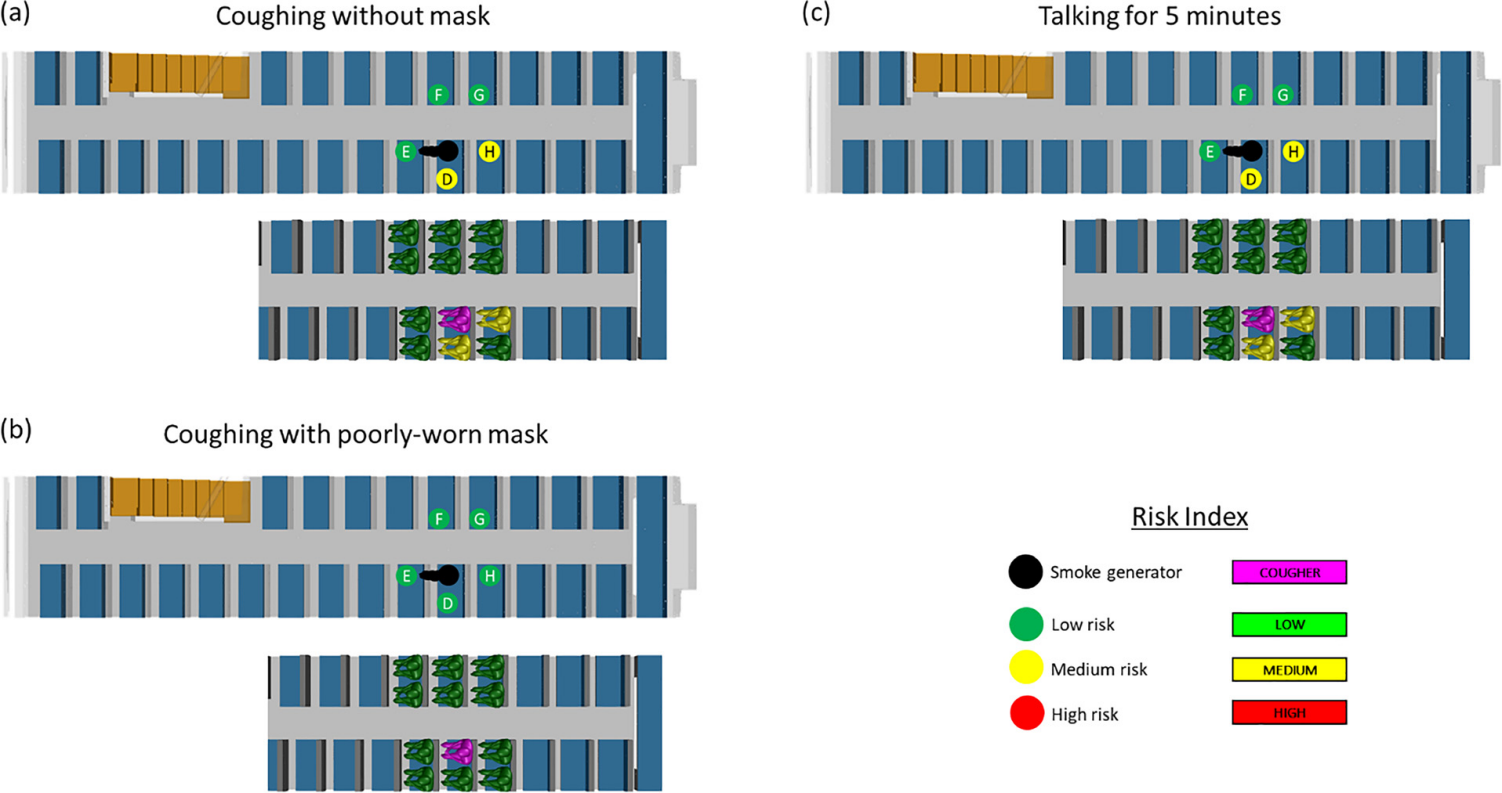
Schematics show the experimental and numerical simulation results for (a) coughing without a mask (case A1); (b) coughing with a poorly worn mask (case A2); and (c) talking for 5 min without a mask (case A3). The top schematic illustrates the results from the surrogate aerosol experiments while the bottom schematic illustrates the results from the CFD simulations. The risk levels are color coded as red, yellow, and green for high, medium, and low risk, respectively, as per the risk bands in Figs. 1 and 2.

In both the CFD simulations and the surrogate aerosol experiments, the impact of having the air-conditioning return at the back of the bus is immediately apparent as it is noticed that the air movement is biased toward the back of the bus. A visualization of the impact of air-conditioning on the air-flow within the bus is provided in the supplementary material, with a clear backward drift of the aerosols when the air-conditioning is turned on (video S2) as compared to off (video S1). In general, the transient simulations also show two distinct trajectories for all simulated cough droplets depending on their initial droplet size. Droplets with diameter larger than 75 *μ*m, i.e., large droplets, tend to be dominated by gravitational and inertial effects and fall relatively near the cougher in the direction of the cough. In contrast, droplets with diameter smaller than 75 *μ*m, i.e., small droplets, are carried by the previously described airflow and are carried toward the back of the bus. An animation of the dispersion of emitted droplets from a cough within the bus is provided in video S3, with a clear backward drift of the smaller aerosols toward the back of the bus. This is also consistent with the literature about the impact of small and large droplet dispersion in COVID-19 transmission and exemplifies previous reports in the literature about the potential for transmission due to airflow in indoor environments.^77^ Figure 7 shows the risk levels associated with each of the scenarios, while the actual results from CFD simulations and surrogate aerosol experiments are provided in Table III.

**TABLE III.** Summary of risk level for cases A1, A2, and A3 based on CFD simulations and surrogate aerosol experiments.

		CFD simulations		Surrogate aerosol experiments	
Scenario		Viral load	Risk level	Relative concentration	Risk level
Coughing w/o mask (A1)	Position D	187	Med	8.5	Med
	Position E	0	Low	4.7	Low
	Position F	0	Low	1.6	Low
	Position G	0	Low	3.3	Low
	Position H	150	Med	7.9	Med
Coughing w/ mask (A2)	Position D	0	Low	3.6	Low
	Position E	0	Low	0.62	Low
	Position F	0	Low	2.0	Low
	Position G	0	Low	2.1	Low
	Position H	0	Low	1.9	Low
Talking w/o mask (A3)	Position D	363	Med	6.3	Med
	Position E	0	Low	2.3	Low
	Position F	12	Low	0.9	Low
	Position G	0	Low	1.5	Low
	Position H	190	Med	6.2	Med

Interestingly, despite the passenger facing forward, a slightly higher risk was observed for the passengers behind and next to the infected individual due to the induced airflow within the bus from the relative supply and return placements, further illustrating the usefulness of such simulations. It is also worth noting that the simulated scenario of coughing with a poorly worn mask still exhibits the lowest risk for all neighboring passengers, which is consistent with current literature on the importance of face masks.^78^

### B. Assessment of relative risk for different locations within the double-decker bus

As a further demonstration of this risk assessment methodology, three separate locations within the double-decker bus are evaluated for the relative risk to nearby passengers in the event of a cough without any face mask. Due to the absence of face masks, the relative risk to neighboring passengers from a coughing infected passenger can be high, and this scenario is hence selected for further study.

**FIG. 8.**
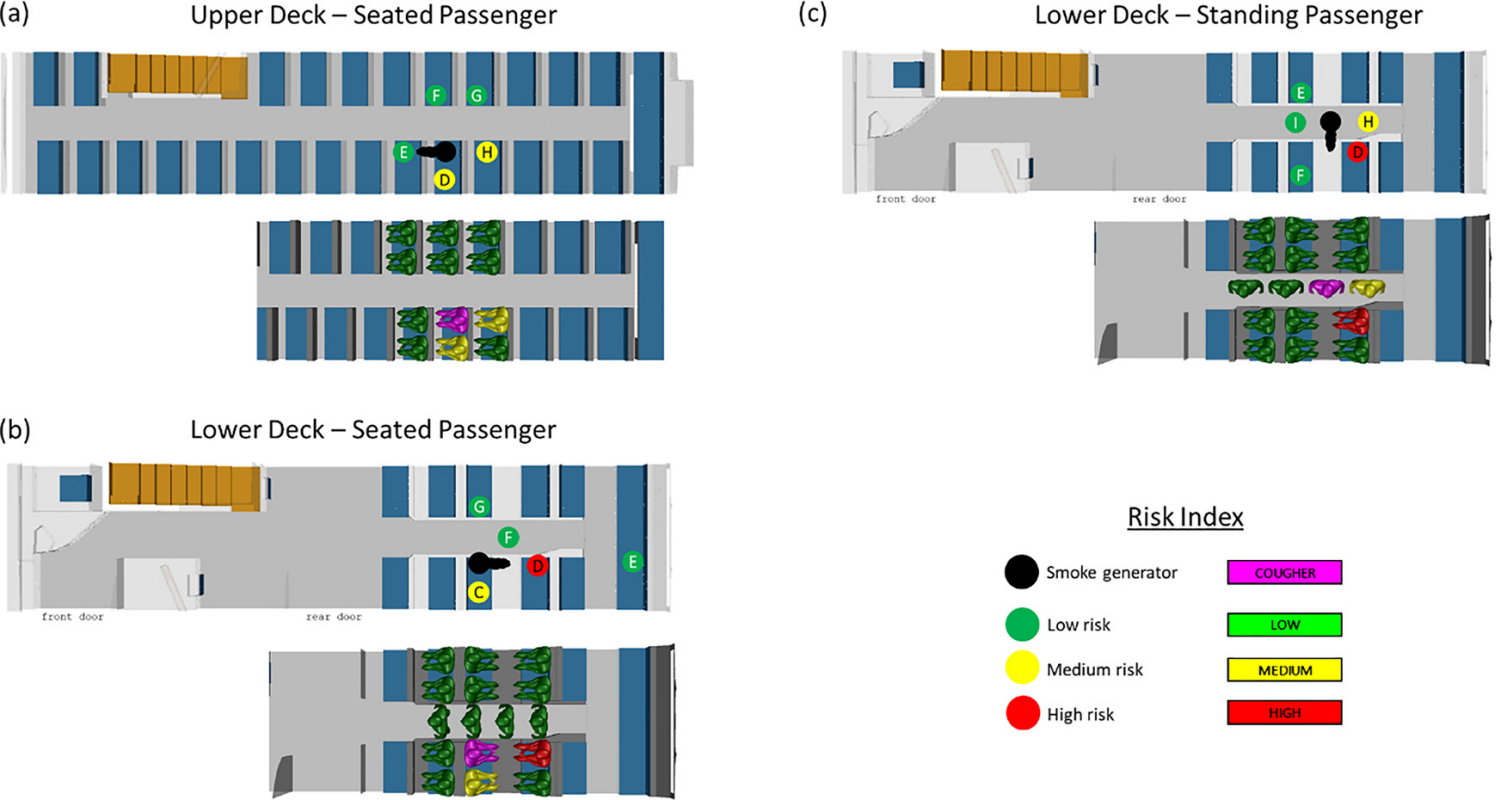
Schematics show the experimental and numerical simulation results for a passenger coughing without a mask while (a) seated on the upper deck (case A1); (b) seated on the lower deck (case B1); and (c) standing on the lower deck (case C1). The top schematic illustrates the results from the surrogate aerosol experiments while the bottom schematic illustrates the results from the CFD simulations. The risk levels are color coded as red, yellow, and green for high, medium, and low risk, respectively, as per the risk bands in Figs. 1 and 2.

In all three scenarios, the distance between individuals alone is not always consistent with the amount of risk exposure. Due to the return duct being situated at the back of the bus, there is medium risk to the passengers behind the cougher for those seated on the upper deck (case A1). A similar trend is observed for all three scenarios in that neighboring passengers down-wind of the cougher are typically at a higher risk than the other passengers. This is particularly true for the standing passenger that is coughing (case C1), as the two passengers next to the individual are also at higher risk. Somewhat surprisingly, the passenger to the side of the coughing seated individual in both upper and lower decks also experiences a slightly higher risk of exposure. Figure 8 shows the risk levels associated with each of the scenarios, while the actual results from CFD simulations and surrogate aerosol experiments are provided in Table IV.

**TABLE IV.** Summary of risk level for cases A1, B1, and C1 based on CFD simulations and surrogate aerosol experiments.

		CFD simulations		Surrogate aerosol experiments	
Scenario		Viral load	Risk level	Relative concentration	Risk level
Upper deck – Seated (A1)	Position D	187	Med	8.5	Med
	Position E	0	Low	4.7	Low
	Position F	0	Low	1.6	Low
	Position G	0	Low	3.3	Low
	Position H	150	Med	7.9	Med
Lower deck – Seated (B1)	Position C	89	Med	15.2	Med
	Position D	163 563	High	48.7	High
	Position E	⋯	⋯	0.6	Low
	Position F	0	Low	0.9	Low
	Position G	0	Low	0.7	Low
Lower deck – Standing (C1)	Position D	2031	High	43.8	High
	Position E	0	Low	1.2	Low
	Position F	0	Low	2.0	Low
	Position H	332	Med	5.1	Med
	Position I	0	Low	1.0	Low

**FIG. 9.**
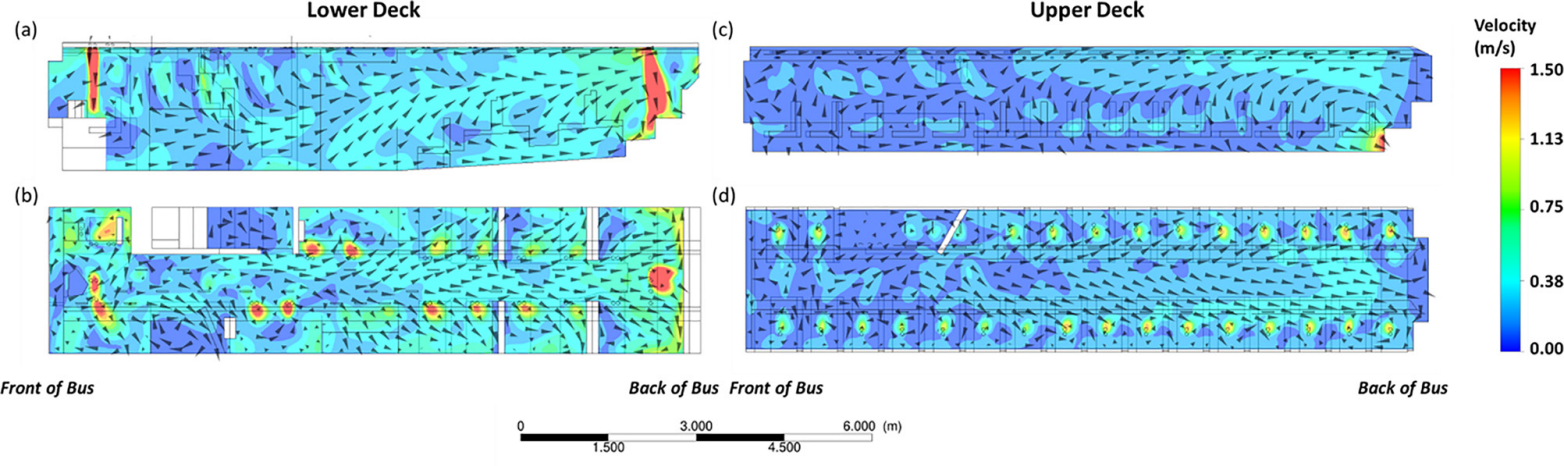
Velocity vector plots from CFD simulations for the lower deck for the (a) vertical and (b) horizontal planes, and the upper deck for the (c) vertical and (d) horizontal planes. The individual plots are all oriented such that the front of the bus is to the left of the figure.

The results can be further understood by reviewing the velocity vectors within the bus as obtained by the CFD simulations. The velocity vectors for both the upper and lower decks show a dominant flow toward the back of the bus. This is consistently observed for both the horizontal and vertical cut-planes across the length of the bus, as per Fig. 9. This further suggests that the results obtained for the individual seat locations as per Fig. 4 can be generalized to different seat locations in the bus.

### C. Comparison between CFD simulations and surrogate aerosol experiments

For the case studies conducted, it is noted from Figs. 7 and 8 that without the use of masks, passengers adjacent to the infected person might have medium to high risk. Hence, in situations where safe distancing might not be feasible such as on buses, it is imperative that passengers maintain the use of face masks. It is also qualitatively apparent that the two different methods provide fairly similar risk assessment results.

As further demonstration of the methodologies' internal consistency, we compute the Spearman's ranked correlation coefficient for the relative aerosol concentration as measured by the sensors and the corresponding calculated viral loads as obtained from CFD for the same locations and obtain a correlation coefficient of 0.74 using the “cor.test” package in R. The Spearman correlation coefficient is a measure of the correlation between the two observables and has a maximum value of 1 when the two variables are monotonically correlated. Hence, a value of 0.74 indicates that the two observables do indeed result in very correlated quantitative results. We also obtained a p-value of 0.000 037, which is far below typical thresholds of p < 0.05 or p < 0.001 used in statistical testing to assess the probability that a statistical result was obtained by chance. This further provides a quantitative basis for the dual use of numerical simulations and experiments, as the high correlation coefficient and low p-value suggest that the two methods are very correlated, and will provide similar results when comparing relative risk across different scenarios under this framework.

## IV. DISCUSSION

Over the course of this pandemic, various authors have noted the need for a more intuitive way of comparing transmission risk and mitigation measures and communicating the results to a less technical audience, including via a decision support framework based on effective rebreathed volume (ERBV).^39^ In this work, we propose the use of a three-tiered risk assessment system to evaluate the potential for COVID-19 transmission across different real-world scenarios and locations. Threshold values are chosen based on the current literature for converting high-fidelity but less interpretable results from both numerical CFD studies and experimental surrogate aerosol studies into a three-tiered risk assessment scale for direct qualitative comparison. This conversion into a simple risk assessment metric can help quantify relative risks across different social settings and mitigation measures as the world emerges from lock-down.

A real-world example of different scenarios within a double-decker air-conditioned bus is then analyzed under this framework to illustrate the implementation of such a methodology for assessing the associated risks. In particular, internal consistency in the results from a joint modeling and experimental approach can provide greater confidence to policy-makers, especially as both CFD modeling and surrogate aerosol dispersion experiments can have certain inadequacies. For example, while CFD can provide high resolution quantitative information such as full flow field and aerosol dispersion dynamics within an entire area of interest, it requires exact specification of boundary conditions, such as ventilation inlet and outlet operating conditions. These values are not always easily obtainable and may not account for real-world imperfections. In contrast, the aerosol dispersion experiments as conducted in the bus can be conducted under actual operating conditions but only provides point measurements of relative reduction in surrogate aerosol concentration. In addition, it can be time-consuming and cost-prohibitive to place sufficient sensors to assess the relative risk across the whole domain, such as in assessing the risk across all seats in a bus. Hence, the joint use of modeling and experiments provides complementary information and independent verification of any findings. In this particular example, the similar findings between both the numerical simulations and experiments with regard to relative risk level of different positions and scenarios within the bus further exemplify how both methods can be used for joint analysis of different scenarios, including the importance of the use of face masks and the strong impact of the internal ventilation system within the bus on aerosol dispersion.

While these proposed methods attempt to simulate the real-world dispersion of aerosols or droplets in realistic settings, there are several caveats. For example, there are simplifying assumption in the specification of droplet size distribution and emission velocities for both the CFD models and the surrogate aerosol experiments. Selected literature-based profiles are assumed to be representative for this work but the extent of inter- or even intra-person variability is currently not quantified. Both methods also assume static humans which may not be accurate in reality. In addition, the information regarding infectivity of COVID-19 is currently very limited; hence, thresholds for the high, medium, and low risk categories were proposed based on a comparison of the estimated minimum infectious dose and estimated safety distances across different authors in literature. This threshold might need adjustment as more accurate information on infectivity surfaces. Nonetheless, the results of this three-tiered assessment are relatively robust to a reasonable choice of threshold values. For example, a choice of 100 and 1000 viral copies, as suggested by Buonanno *et al.* and Mittal *et al.*,^22,34^ would not change the relative risk levels for 96% of the locations studied across the different scenarios. Other complex effects such as the impact of turbulent fluctuations on the dispersion of the particles are also worth studying in greater detail in future work. This is a complicated phenomenon, where different models can potentially affect the estimate of viral load that lands on the individual. Nonetheless, when we compared the results for case A1 between simulations with and without a discrete random walk model to account for turbulent dispersion, we noticed that the relative risk levels did not change for the individuals as well. The impact of turbulent dispersion could however vary significantly across different scenarios. Other factors such as the risk of fomite transmission which incorporate other complexities such as surface stability of SARS-CoV-2 are also not accounted for in this current methodology.^79^

Nonetheless, as a risk assessment methodology, results from the use of a consistent, single simulation parameter across different scenarios can still provide valuable information for decision making. As suggested by Mittal *et al.*, a consistent framework for estimating airborne transmission risk can at least serve as a means of quantifying relative risk or risk reduction across different scenarios, even in the presence of uncertainty over specific model parameters.^40^ It is also worth noting that several of these model parameters can be easily adjusted for the extension of this proposed methodology to the study of other infectious diseases in the future with better-defined epidemiology. The dual use of numerical CFD simulations and surrogate aerosol experiments can compensate for assumptions specific to each methodology and provide greater confidence in the results obtained, even while the simplified risk assessment system is more easily interpreted.

## SUPPLEMENTARY MATERIAL

See the supplementary material for the forward motion of surrogate aerosols when the air-conditioning is not turned on in the bus as obtained from experiments (Video S1), the backward motion of surrogate aerosols when the air-conditioning is turned on in the bus as obtained from experiments (Video S2), and the backward motion of emitted droplets in the presence of air-conditioning in the bus as obtained from CFD simulations (Video S3).

## Data Availability

The data that support the findings of this study are available from the corresponding author upon reasonable request.
